# Soluble CD14 and Incident Diabetes Risk: The REasons for Geographic and Racial Differences in Stroke (REGARDS) Study

**DOI:** 10.1210/jendso/bvae097

**Published:** 2024-05-23

**Authors:** Kaileen Cruden, Katherine Wilkinson, Debora Kamin Mukaz, Timothy B Plante, Neil A Zakai, D Leann Long, Mary Cushman, Nels C Olson

**Affiliations:** Larner College of Medicine at the University of Vermont, Burlington, VT 05405, USA; Department of Pathology and Laboratory Medicine, Larner College of Medicine at the University of Vermont, Burlington, VT 05405, USA; Department of Medicine, Larner College of Medicine at the University of Vermont, Burlington, VT 05405, USA; Department of Medicine, Larner College of Medicine at the University of Vermont, Burlington, VT 05405, USA; Department of Pathology and Laboratory Medicine, Larner College of Medicine at the University of Vermont, Burlington, VT 05405, USA; Department of Medicine, Larner College of Medicine at the University of Vermont, Burlington, VT 05405, USA; Department of Biostatistics, School of Public Health, University of Alabama at Birmingham, Birmingham, AL 35233, USA; Department of Pathology and Laboratory Medicine, Larner College of Medicine at the University of Vermont, Burlington, VT 05405, USA; Department of Medicine, Larner College of Medicine at the University of Vermont, Burlington, VT 05405, USA; Department of Pathology and Laboratory Medicine, Larner College of Medicine at the University of Vermont, Burlington, VT 05405, USA

**Keywords:** biomarkers, diabetes, inflammation, racial groups

## Abstract

**Context:**

Soluble CD14 (sCD14) is an inflammation biomarker with higher concentrations in White than Black adults. Higher sCD14 is seen in insulin resistance and diabetes. There are limited data on the relationship between sCD14 and incident diabetes.

**Objective:**

To determine the association of sCD14 with incident diabetes risk in a large biracial US cohort and evaluate whether relationships differ by race.

**Design:**

This study included 3401 Black and White participants from the REasons for Geographic And Racial Differences in Stroke (REGARDS) study without baseline diabetes who completed baseline and follow-up in-home visits. Modified Poisson regression models estimated risk ratios (RR) of incident diabetes per 1-SD increment sCD14, with adjustment for risk factors. A sCD14-by-race interaction evaluated whether associations differed by race.

**Results:**

There were 460 cases of incident diabetes over a mean 9.5 years of follow-up. The association of sCD14 with diabetes differed by race (*P* for interaction < .09). Stratifying by race, adjusting for age, sex, and region, higher sCD14 was associated with incident diabetes in White (RR: 1.15; 95% CI: 1.01, 1.33) but not Black participants (RR: 0.96; 95% CI: 0.86, 1.08). In models adjusted for clinical and sociodemographic diabetes risk factors, the association was attenuated among White participants (RR: 1.10; 95% CI: 0.95, 1.28) and remained null among Black participants (RR: 0.90; 95% CI: 0.80, 1.01).

**Conclusion:**

sCD14 was associated with incident diabetes risk in White but not Black adults, but this association was explained by diabetes risk factors.

Cluster of differentiation 14 (CD14) is a pattern recognition receptor that plays roles in the inflammatory response to bacterial cell wall components, such as lipopolysaccharide (LPS), and endogenous macromolecules including heat shock proteins and phospholipids [1]. Membrane CD14 is attached to the plasma membrane of monocytes, macrophages, neutrophils, and other immune cells by a glycosylphosphatidylinositol anchor. A soluble form of CD14 (sCD14) circulates following hepatic secretion during an acute phase response and cell shedding or cleavage of the membrane-bound form. Systemic sCD14 concentrations increase with acute and chronic inflammation [2] and blood levels vary by self-reported race, with higher concentrations in White than Black adults [3, 4].

sCD14 has been implicated as a risk factor for cardiometabolic diseases [1, 2]. In several observational cohort studies, higher sCD14 concentration was related to incident cardiovascular disease (CVD) events independent of traditional risk factors and other inflammation biomarkers [3, 5-7]. The REasons for Geographic and Racial Differences in Stroke (REGARDS) study demonstrated higher sCD14 level was associated with increased risks of coronary heart disease and ischemic stroke in Black participants but not White participants [4]. Like these vascular conditions, Black adults in the United States experience a disproportionate burden of type 2 diabetes mellitus [8].

While experimental animal models have demonstrated a role of CD14 signaling in insulin resistance [9, 10], limited data are available on this question from human studies. Higher sCD14 concentration was associated with prevalent diabetes in 4 cross-sectional studies [3, 5, 11], including REGARDS [4]. To our knowledge, only one study, comprising Black and White adults aged 65+, examined the relationship of sCD14 with incident diabetes risk [11], and observed no association with incident diabetes. This study did not report associations separately by race.

We consider race as a social construct reflecting factors like structural racism and historic differences in socioeconomic status and other factors [12]. We hypothesized the relationship of sCD14 with incident diabetes risk may differ by race [3, 4, 13]. We approached this question by evaluating the association of sCD14 with risk of incident diabetes in Black and White participants of the REGARDS Biomarkers as Mediators of racial disparities in Risk factors (BioMedioR) nested cohort study.

## Methods

### Cohort

REGARDS is a longitudinal cohort study of 30 239 Black and White adults aged 45 and older [14]. Participants were enrolled across the contiguous United States between 2003 and 2007. The cohort, by design, was 41% Black and 55% women, and 56% resided in the US “Stroke Belt” (AL, AR, GA, LA, MS, NC, SC, and TN) and “Stroke Buckle” (coastal plain of NC, SC, and GA). Participants underwent a computer-assisted telephone interview (CATI) assessing demographic, lifestyle, and CVD risk factor information [14]. At baseline (2003-2007) and follow-up (2013-2016) examinations, participants received in-home visits for assessment of anthropomorphic measures and vascular disease risk factors, and to collect fasting blood samples [14, 15].

BioMedioR is a nested cohort study within REGARDS designed to determine the reasons underlying racial disparities in hypertension and diabetes incidence [16]. BioMedioR included a race- and sex-stratified cohort random sample of participants (n = 4400) attending both in-home visits. Participants provided written informed consent for study participation. Procedures and protocols were approved by Institutional Review Boards.

### Laboratory Methods and Definitions

sCD14 was measured by Human CD14 Quantikine ELISA (R&D Systems, Inc., Cat# DC140, RRID:AB_3095885) using fasting serum collected at baseline and stored at −80°C. The assay coefficient of variation was 12.0%. Laboratory measurements for serum glucose, high-density lipoprotein (HDL), and triglycerides were performed using an Ortho Vitros analyzer (Johnson & Johnson Clinical Diagnostics, Inc.).

Sex, race, and medication use data were obtained by participant self-report. Education was categorized as less than high school, high school degree, some college, or college degree and above. Annual household income was categorized as < $20 000, $20 000-$35 000, $35 000-$75 000, and ≥ $75 000 with a separate category for those unwilling to disclose this information. Physical activity was defined as none, 1 to 3 times per week, or 4 or more times per week.

Body mass index (BMI) categories were defined as normal (18.5-24.9 kg/m^2^), overweight (25.0-29.9 kg/m^2^) and obese (30.0 + kg/m^2^). Diabetes was defined as a fasting glucose level ≥ 126 mg/dL, a nonfasting glucose ≥ 200 mg/dL, or by participant self-report of diabetes medication use. Incident diabetes was defined as being free of diabetes at the first in-home visit with diabetes at the second in-home visit. REGARDS did not differentiate type 1 from type 2 diabetes. As the incidence of type 1 diabetes in North America is estimated as 20 per 100 000, it is expected that diabetes cases in REGARDS are nearly exclusively type 2 [17].

### Statistical Analysis

Among the 4400 participants in BioMedioR, 930 with prevalent diabetes and 69 without sCD14 data were excluded, leaving 3401 participants for analysis. Associations of baseline sCD14 with risk of incident diabetes were estimated by risk ratios (RR) calculated using Poisson regression with robust standard error estimation [18]. sCD14 was analyzed as a continuous variable per 1-SD increment. Models included sampling weights to account for BioMedioR's stratified sampling design and reflect the entire REGARDS cohort who attended both in-home visits [16].

Models included 4 tiers of risk factor adjustment. Model 1 adjusted for age, sex, race, and region (demographic model). Model 2 added adjustment for BMI category. Model 3 added the clinical risk factors, triglycerides, HDL cholesterol, statin use, systolic blood pressure, and hypertension medication use to Model 2. Model 4 included additional demographic and lifestyle risk factors (Model 3 plus education, income, and physical activity [fully adjusted risk factor model]). In post hoc analyses, we adjusted Model 3 for either lipid or blood pressure measures, separately, to determine which clinical risk factors attenuated the sCD14 association.

We tested sCD14-by-race and sCD14-by-sex interaction terms a priori to assess for differences in the relationships between sCD14 and diabetes risk by self-reported racial group and by self-reported sex. Statistical significance for the interaction term was defined as *P* < .10 [19]. Sensitivity analyses assessed nonlinear relationships of sCD14 with diabetes using restricted cubic spline models and adjusted for waist circumferences in place of BMI category.

## Results

Table 1 shows the baseline characteristics of the BioMedioR study population by sCD14 quartile. There were 460 cases of incident diabetes over a mean follow-up time of 9.5 years (278 cases in Black and 182 in White participants). In testing for interactions, the interaction term for sCD14-by-race, but not sCD14-by-sex (*P* value in all models > .10), was statistically significant. Since the sCD14-by-race interaction term was significant, analyses were stratified by race.

**Table 1.** Baseline characteristics of the REGARDS BioMedioR cohort by sCD14 quartiles, weighted to account for sampling design

Variable	Overall, N (%) or mean (SD)	sCD14			
		Q1	Q2	Q3	Q4
Sampled cohort	3401	878	851	844	828
Weighted cohort	11 081	2773	2771	2769	2768
Age (years)	63 (8.3)	62.1 (8.2)	62.8 (8.3)	63.3 (8.4)	63.7 (8.3)
Male	4938 (45%)	1528 (55%)	1315 (47%)	1168 (42%)	927 (33%)
Female	6143 (55%)	1244 (45%)	1456 (53%)	1602 (58%)	1841 (67%)
Black	3551 (32%)	885 (32%)	889 (32%)	889 (32%)	888 (32%)
White	7530 (68%)	1880 (68%)	1883 (68%)	1881 (68%)	1886 (68%)
Region					
Stroke belt or stroke buckle	6073 (55%)	1551 (56%)	1589 (57%)	1520 (55%)	1413 (51%)
Rest of United States	5008 (45%)	1221 (44%)	1183 (43%)	1249 (45%)	1355 (49%)
Less than high school education	779 (7%)	186 (7%)	161 (6%)	183 (7%)	249 (9%)
Income					
< $35k	3592 (32%)	762 (28%)	862 (32%)	958 (35%)	983 (35%)
Not disclosed	1198 (11%)	286 (10%)	257 (9%)	296 (11%)	359 (13%)
Physical activity (weekly)					
None	2999 (27%)	749 (27%)	700 (25%)	795 (29%)	755 (27%)
1-3 times	4253 (38%)	1078 (39%)	1070 (39%)	1062 (38%)	1043 (38%)
≥ 4 times	3692 (33%)	912 (33%)	977 (35%)	867 (31%)	936 (34%)
BMI (kg/m^2^)	11 054 (99.7%)	28.6 (5.1)	28.8 (5.5)	28.7 (5.7)	28.8 (5.8)
Systolic blood pressure (mm Hg)	124 (15)	124 (15)	124 (14)	125 (15)	125 (16)
Blood pressure medication use	4539 (41%)	1071 (39%)	1079 (39%)	1160 (42%)	1229 (44%)
HDL cholesterol (mg/dL)	53 (16)	52 (16)	53 (16)	54 (16)	55 (17)
Triglycerides (mg/dL)	125 (83)	125 (90)	126 (74)	123 (70)	128 (94)
Statin use	2898 (26%)	911 (33%)	605 (22%)	733 (26%)	649 (23%)
Incident diabetes unweighted (N)*^a^*	460 (13%)	121 (14%)	122 (14%)	100 (12%)	117 (14%)
Incident diabetes weighted (N)	1373 (12%)	335 (13%)	362 (13%)	304 (11%)	372 (13%)

sCD14 was evaluated as race-specific quartiles (Q) of the sCD14 distribution: White participants: Q1: ≤ 1217 pg/mL; Q2: 1218-1395 pg/mL; Q3: 1396-1609 pg/mL; Q4: > 1609 pg/mL. Black participants: Q1: ≤ 1112 pg/mL; Q2: 1113-1292 pg/mL; Q3: 1293-1515 pg/mL; Q4: > 1515 pg/mL.

^*a*^The unweighted sample size reflects the number of BioMedioR participants with incident diabetes included in the analysis.

Adjusting for demographic factors, each 1-SD increment sCD14 (331 pg/mL) was associated with a higher risk of incident diabetes in White participants (RR: 1.15; 95% CI: 1.01, 1.33) and a nonsignificant lower risk in Black participants (RR: 0.96; 95% CI: 0.86, 1.08) (*P*-interaction = .09). Results were similar following adjustment for BMI (Model 2 RR [95% CI] White participants: 1.19 [1.04, 1.36]; Black participants: 0.98 [0.97, 1.00]; *P*-interaction = .05). In models adjusted for additional clinical risk factors (Model 2 + triglycerides, HDL cholesterol, statin use, systolic blood pressure, and hypertension medication use), the association of sCD14 with diabetes was attenuated to null among White participants (Model 3 RR: 1.12; 95% CI: 0.97, 1.28) and remained nonsignificant among Black participants (Model 3 RR: 0.94; 95% CI: 0.84, 1.06) (*P*-interaction = .05). In the fully adjusted model that included additional demographic and lifestyle factors associations were also null (Model 4 RR [95% CI] White participants: 1.10 [0.95, 1.28]; Black participants: 0.90 [0.80, 1.01]; *P*-interaction = .03).

Guided by the results from Model 3 which suggested adjustment for lipid or blood pressure measures attenuated sCD14's association with diabetes, we performed post hoc analyses adjusting Model 3 separately for either lipid or blood pressure variables. In models adjusted for age, sex, region, BMI category, triglycerides, HDL cholesterol, and statin use the sCD14 association with diabetes risk was statistically significant in White (RR: 1.18, 95% CI: 1.03, 1.34), but not Black participants (RR: 0.95, 95% CI: 0.85, 1.07) (*P*-interaction .03). In a model replacing lipids with systolic blood pressure and hypertension medication use, the sCD14 association was null in both race groups (RR [95% CI] White participants: 1.13 [95% CI: 0.98, 1.30]; Black participants: 0.96 [95% CI: 0.86, 1.08]; *P*-interaction .09).

**Figure 1.**
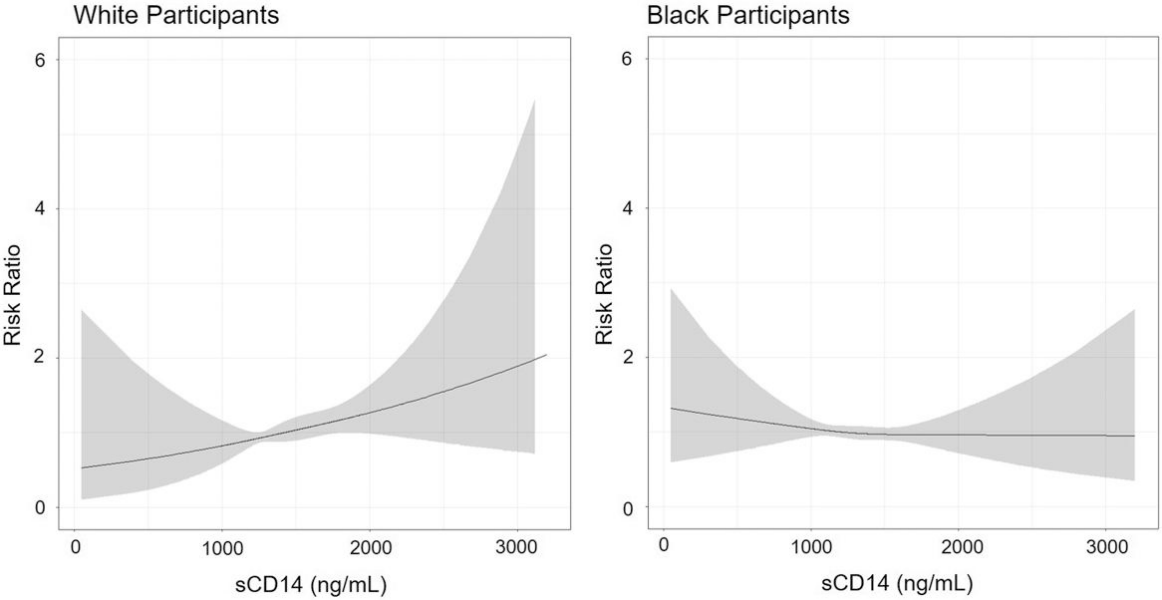
Restricted cubic spline modeling of sCD14 with incident diabetes risk stratified by race. Solid lines represent risk ratios; shaded areas represent 95% confidence intervals. Knots were race-specific, located at 944, 1281, and 1726 pg/mL with the reference category set at 1292 pg/mL among Black participants. Among White participants knots were located at 1068, 1395, and 1829 pg/mL with the reference set at 1395 pg/mL. Analyses adjusted for age, sex, and region.

In sensitivity analyses, we did not observe any departures from linearity using restricted cubic spline models (Fig. 1). Adjusting for waist circumference in place of BMI category in Models 2 to 4 did not alter interpretation of the results.

## Discussion

In this prospective biracial US cohort study, higher sCD14 was associated with incident diabetes risk in White but not Black adults. This association was explained by diabetes risk factors and there was no evidence of a nonlinear association.

Several prior studies have observed relationships between higher sCD14 concentration and prevalent insulin resistance and diabetes [3-5, 20, 21]. Whether a higher sCD14 level precedes diabetes onset or is a cause of the disease, however, has only been investigated in one study to our knowledge, the older-age Cardiovascular Health Study (CHS), comprising adults aged 65+ [11]. The null results from REGARDS are consistent with those from CHS, which did not observe an association of sCD14 protein concentration with incident diabetes risk [11], although associations by racial group were not presented. However, for sCD14 genetic variation and insulin resistance, race-stratified Mendelian randomization analyses performed in CHS demonstrated that sCD14-associated single nucleotide polymorphisms were not associated with prevalent insulin resistance in self-reported Black or White participants [11]. Although race is an inadequate proxy for genetic ancestry because it reflects historical and socio-political processes and not biological divisions of human variations [22], the null Mendelian randomization results in CHS also align with our results for incident diabetes in REGARDS.

Three prior epidemiological cohort studies, including REGARDS, have implicated sCD14 as a CVD risk factor [3-5]. CD14 is proposed to contribute to CVD risk by promoting chronic, low-grade inflammation in response to stimulation from LPS during metabolic endotoxemia and from low-density lipoprotein metabolites (LDL), including oxidized LDL [1]. In this context, CD14 signaling has been shown to play roles in foam cell formation, macrophage and coronary artery smooth muscle cell proinflammatory cytokine secretion, and endothelial cell adhesion molecule expression [23-26], which are putative mechanisms linking CD14 to CVD.

Since chronic inflammation is an established risk factor for insulin resistance and type 2 diabetes, we hypothesized that CD14 inflammatory signaling would be associated with increased risk of diabetes onset. This hypothesis was supported by results from animal models whereby CD14-deficient mice were protected from insulin resistance and glucose intolerance when fed a high fat diet [9, 10]. CD14's roles in metabolism, however, appear complex. Administration of sCD14 to obese mice improved glucose tolerance by downregulating the response of membrane-bound CD14 to LPS [9]. Other studies have reported that CD14-deficient mice have impaired glucose tolerance at older ages compared with wild-type [27]. Further, CD14 has been suggested to play additional roles in metabolic diseases besides inflammation. CD14 was reported to regulate glucose tolerance by suppressing the sympathoadrenal axis [10, 27]. CD14 expression in pancreatic islets was suggested to monitor the endocrine environment and modulate glucose-dependent insulin release [28, 29].

Overall, the potential mechanisms underlying differences in the associations of sCD14 with incident CVD events and incident diabetes observed in REGARDS and other studies require further study. Collectively, results suggest that while sCD14 level may be related to the presence of diabetes, it is not a risk factor for disease onset [3-5, 11, 20].

The differential association of sCD14 with incident diabetes by racial group in models adjusted for demographic and adiposity variables, with a higher risk in White adults and no association in Black adults, was counter to our hypothesis. Prior results in REGARDS indicated that although sCD14 blood concentrations were significantly lower in Black than White participants, higher sCD14 was associated with coronary heart disease and ischemic stroke risk among Black but not White adults [4]. The association among White participants in the current study was attenuated following adjustment for blood pressure variables, although a statistically significant sCD14-by-race interaction was observed in all models tested. Potential reasons underlying the differential association by self-reported race are unclear. Results may be due to complex and incompletely understood interrelationships of sCD14 with metabolism pathways [2, 30] or other diabetes-related factors that differ by race. We also cannot rule out the possibility of chance findings. Overall, results suggest sCD14 does not biologically mediate racial disparities in the risk of diabetes and the observed differential association by race group is unlikely to have clinical implications.

Limitations of this study merit attention. In BioMedioR there was a higher prevalence of diabetes in Black than White participants at the baseline REGARDS visit. This resulted in unequal proportions of Black and White people excluded from the analyses and a smaller number of Black participants at risk for incident diabetes during follow-up. We did not assess some structural determinants of health which disproportionately promote diabetes risk among Black people [31]. Strengths of this study are the inclusion of a nationally representative sample with representation of Black and White individuals and a large number of incident diabetes cases.

In conclusion, sCD14 was associated with incident diabetes in White but not Black adults but this association was explained by traditional diabetes risk factors and is unlikely to have clinical implications.

## Data Availability

Restrictions apply to the availability of some or all data generated or analyzed during this study to preserve patient confidentiality or because they were used under license. The corresponding author will on request detail the restrictions and any conditions under which access to some data may be provided.

## Abbreviations

BioMedioR: REGARDS Biomarkers as Mediators of racial disparities in Risk factors nested cohort study
BMI: body mass index
CD14: cluster of differentiation 14
CHS: Cardiovascular Health Study
CVD: cardiovascular disease
HDL: high-density lipoprotein
LDL: low-density lipoprotein
LPS: lipopolysaccharide
sCD14: soluble form of CD14
REGARDS: REasons for Geographic and Racial Differences in Stroke
RR: risk ratio
